# A CMOS-Compatible Poly-Si Nanowire Device with Hybrid Sensor/Memory Characteristics for System-on-Chip Applications

**DOI:** 10.3390/s120403952

**Published:** 2012-03-26

**Authors:** Min-Cheng Chen, Hao-Yu Chen, Chia-Yi Lin, Chao-Hsin Chien, Tsung-Fan Hsieh, Jim-Tong Horng, Jian-Tai Qiu, Chien-Chao Huang, Chia-Hua Ho, Fu-Liang Yang

**Affiliations:** 1 National Nano Device Laboratories, No. 26, Prosperity Road 1, Hsinchu Science Park, Hsinchu 300, Taiwan; E-Mails: chenhys@tsmc.com (H.-Y.C.); cylin@ndl.narl.org.tw (C.-Y.L.); cchuang@ndl.narl.org.tw (C.-C.H.); chho@ndl.narl.org.tw (C.-H.H.); flyang@ndl.narl.org.tw (F.-L.Y.); 2 Institute of Electronics, National Chiao Tung University, Hsin-Chu 300, Taiwan; E-Mail: chchien@faculty.nctu.edu.tw; 3 Graduate Institute of Biomedical Sciences, Chang Gung University, Taoyuan 333, Taiwan; E-Mails: triky333@yahoo.com.tw (T.-F.H.); jimtong@mail.cgu.edu.tw (J.-T.H.); jtqiu@mail.cgu.edu.tw (J.-T.Q.)

**Keywords:** nano-sensor fabrication, nanowire FET, nonvolatile memories, semiconductive sensors

## Abstract

This paper reports a versatile nano-sensor technology using “top-down” poly-silicon nanowire field-effect transistors (FETs) in the conventional Complementary Metal-Oxide Semiconductor (CMOS)-compatible semiconductor process. The nanowire manufacturing technique reduced nanowire width scaling to 50 nm without use of extra lithography equipment, and exhibited superior device uniformity. These n type polysilicon nanowire FETs have positive pH sensitivity (100 mV/pH) and sensitive deoxyribonucleic acid (DNA) detection ability (100 pM) at normal system operation voltages. Specially designed oxide-nitride-oxide buried oxide nanowire realizes an electrically V_th_-adjustable sensor to compensate device variation. These nanowire FETs also enable non-volatile memory application for a large and steady V_th_ adjustment window (>2 V Programming/Erasing window). The CMOS-compatible manufacturing technique of polysilicon nanowire FETs offers a possible solution for commercial System-on-Chip biosensor application, which enables portable physiology monitoring and *in situ* recording.

## 1. Introduction

Nanotechnology is being widely investigated in biosensor development to supplant traditional chemical biomolecules detection [1,2]. Different sensitive methods, including optical-based detection (surface plasmon resonance), mechanically based detection (cantilevers), and electrical-based detection (capacitive electrode and nanowire device) are attractive in a number of biomolecule application fields [1–7]. Among them, semiconducting nanowire FET sensors [4] have a great potential to function as label-free, highly accurate, and real-time detectors of low concentrations of proteins [5,8], viruses [6], and DNA [7,9]. Compared to other detection methods, the detection functionality of a nanowire sensor is verified through pure electrical signal characterization; therefore, neither special devices nor materials are necessary. Thus, the production costs could potentially be reduced compared to other sensors. In certain studies, semiconductor nanowires were prepared by the “bottom-up” process [4], which leads to several commercialization problems because of the difficulties of positioning individual nanowires. Consequently, various “top-down” processes for fabricating silicon nanowires have been proposed to provide a solution for manufacturing reliable biosensors because of its compatibility with current commercial silicon-based CMOS technology [6,10]. However, to obtain a large surface-to-volume ratio nanowire with extraordinary detection sensitivity, special techniques or advanced lithography tools were employed to achieve the slender nanowire patterns [10,11]. Therefore, determining an approach on the quick fabrication of a large amount of reliable devices, controlling the electrical properties response at relatively simple detection, and reducing productive costs in commercial scale will be an important issue for using silicon nanowire FETs in future biomedical applications [12].

Table 1 shows a comparison of the Si nanowire FET fabrication in previous studies [4–6,10–14] and this work. Most semiconductor nanowires composed using the “bottom-up” process [4–6] display good detection performances, but are difficult to mass produce and integrate with the CMOS process. Moreover, the “top-down” process [10,13–15] requires advanced exposed equipment (e-beam lithography) or a special pattern design to improve nanowire uniformity and sensitivity. Although numerous studies have been performed for improving nanowire detection sensitivity [14], CMOS circuit integration with signal processing and data storing remains deficient. In this study, a System-on-Chip biosensor was developed using “top-down” poly-Si nanowire FETs in the conventional CMOS-compatible process. To embed nanowire FETs at the back end of line (BEOL) stage of a VLSI circuit, the fabrication of thin-film transistors with poly-Si nanowire channels were employed for low-cost semiconductor manufacturing [16]. With no expensive lithography tools and with desirable process compatibility, the poly-Si nanowire sensor fabrication is favorable for traditional CMOS integration. Furthermore, the electrical characteristics of the nanowire devices can be adjusted by programming or erasing the nitride charge of the specially designed oxide-nitride-oxide (ONO)-buried oxide [17]. This embedded EEPROM cell can be integrated easily into the sensor circuit. The combination of sensor, memory, and circuit in the CMOS-compatible process provides a system-scale integration solution of smart biosensor application for low-cost commercial manufacturing [18].

## 2. Samples Preparation

**Figure 1.**
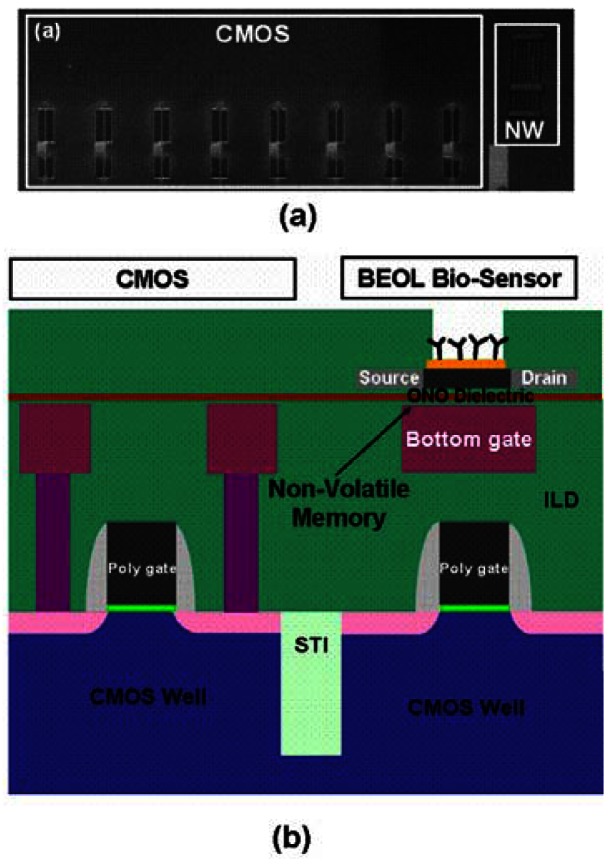
(**a**) Top-View SEM image of the nanowire and CMOS homogenous integration; (**b**) Cross-sectional schematic diagram of the hybrid technology with CMOS devices and biosensor embedded with memory functionality.

**Figure 2.**
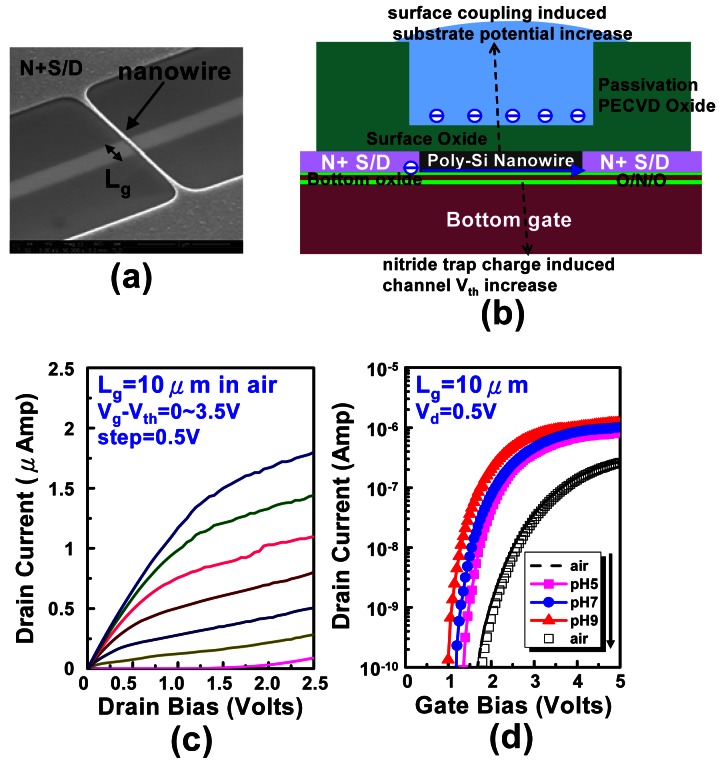
(**a**) Top-View SEM image of the poly-Si nanowire FETs; (**b**) Schematic representation of the bottom gate poly-Si nanowire FETs cross-sectioned with pH solution; (**c**) I_d_-V_d_ output characteristics of the poly-Si nanowire FET device measured in air; (**d**) I_d_-V_g_ characteristics of the poly-Si nanowire FETs measured in air/aqueous pH solution. The pH testing operation did not degrade the electrical characteristics of the device.

The device samples were manufactured on standard 6-in. p-type wafers. A proposed hybrid sensor/memory/CMOS poly-Si nanowire structure is illustrated in Figure 1(b). The bottom-gate poly-Si nanowire formation can be inserted specifically after metallization of the back-end process (BEOL). At the beginning, buried oxide was deposited on a substrate surface as the gate dielectric of nanowire FETs. A 50-nm polysilicon layer was then deposited using the CVD process. Subsequently, the poly-Si wire was patterned by the standard I-line stepper of the CMOS semiconducting process. By using reactive plasma etching for photoresist trimming followed by silicon etching, the nanowire dimension was scaled to a level of approximately 100 nm. A nanowire shrinkage technique using poly re-oxidation and oxide stripping was employed to scale down the nanowire width to less than 50 nm. A channel protection photoresist pattern was then formed by I-line lithography. The objective of the channel protection patterning was to keep the channel intrinsically from n+ source/drain (S/D) implantation, to increase nanowire FETs sensitivity. Subsequently, the n+ S/D implant was performed with a 10^15^ cm^−2^ P^31+^ ion beam at 10 keV to reduce the parasitic resistance of the nanowire. Thereafter, the channel protection photoresist was removed. Finally, the S/D dopant was activated by annealing treatment at 600 °C for 30 min in a N^2^ ambience. The top-view SEM image of the hybrid sensor/memory/CMOS circuit is shown in Figure 1(a). The fabrication flow requires only two extra masks and can be integrated into a standard BEOL process. Figure 2(a) shows the SEM image of the nanowire devices, and Figure 2(b) illustrates the operation mode diagram of the bottom-gate poly-Si nanowire device for ionic solution detection and nitride trap charge storing. Figure 2(c,d) plot the I_d_-V_g_ and I_d_-V_d_ characteristics of the poly-Si nanowire FETs. The device on-off ratio is approximately 10^5^, and the subthreshold swing is 0.5 V/dec. The device threshold voltage was defined by the gate bias at a constant current:

(1) $${\text{I}}_{\text{d}}=10^{-7}\times \frac{L_{g}}{W}$$

where the drain bias, V_d_, is 0.5 V, L_g_ is nanowire channel length, and W is the nanowire width.

## 3. Experimental Characteristics

**Figure 3.**
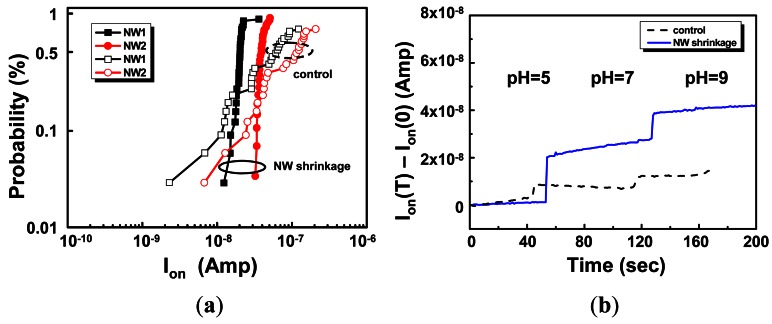
(**a**) The current distribution of various wire widths. The nanowire shrinkage technique can effectively improve the surface roughness-induced variability after PR trimming; (**b**) Real-time current data with sequential pH testing. The sensors of the nanowire shrinkage technique have more obvious pH sensitivity.

The mass manufacture of the semiconductors can simultaneously hold 32 dies in each 6-in. wafer. Figure 3(a) shows the statistics of a comparison among the electrical characteristics of various wire widths without and with the nanowire shrinkage process. The driving current I_on_ is measured, with a bottom gate of 5 V and a drain bias of 0.5 V, without any fluid on the nanowire surface. After the shrinkage process, the statistical plot shows less variation tailing for the improvement of PR trimming-induced surface roughness. Because device-to-device variation can be controlled significantly using the shrinkage process, pH sensitivity testing or memory characteristics in this paper are completed for individual nanowire devices, for fair comparison. On the side, the surface adhesion stress of fluidic cell spatial deflection [19] can also be aligned for nanowire structure uniformity. Figure 3(b) displays I_on_ *versus* time data as phosphate buffer solutions, with pH levels of 5, 7, and 9, which were sequentially delivered onto the nanowire sensors without surface treatment. The nanowire shrinkage split has a high I_on_ change caused by the large surface-to-volume ratio. The functionality of the poly-Si nanowire sensor can improve I_on_ accumulation distribution, and further enhance electrical pH sensitivity. Thus, the pH sensors of the poly-Si nanowire FETs not only have a higher manufacturing production yield, but also have a greater tolerance for the signal-to-noise ratio [20], for future single-chip system integration of nano-sensors.

## 4. Results and Discussion

### 4.1. Nanowire for Biosensor Application

**Figure 4.**
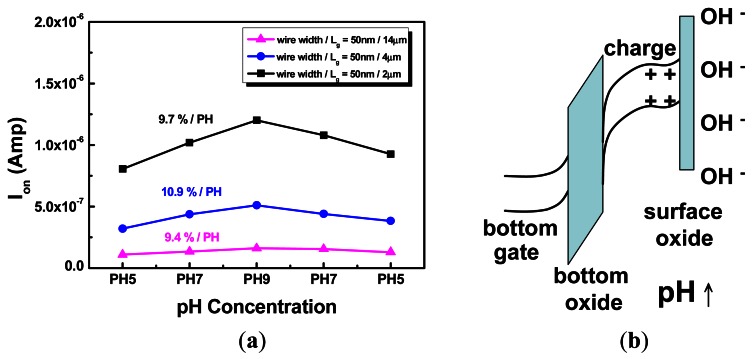
(**a**) pH sensitivity of n-type poly-Si nanowire FETs with pH value increases, from 5, 7, to 9, and sequentially in reverse to 7 and 5. The positive pH sensitivity can be reversible after sequential pH testing; (**b**) Band diagrams explaining the changes in the I_d_-V_g_ characteristics after changing the pH solutions. A high pH solution increases the positive potential and lowers the potential barrier of the nanowire channel substrate.

Figure 2(d) also shows the I_d_-V_g_ curves of the n-type poly-Si nanowire FETs in different pH solutions flowing without surface treatment. The testing sequence is indicated by the arrow. No I-V degradation is present after pH testing, and it displays an obvious V_th_ decrease and I_on_ increase with a rising pH value. The superior pH sensitivity (>100 mV/pH) is an opposite property and beyond the Nernst limitation (60 mV/pH) to ion-selective FETs [21,22]. The I_on_ increases and returns to its original value as the pH value rises from 5, 7, and 9, and reverses sequentially (Figure 4(a)). Consequently, the positive current shifts (10%/pH) are repeatable in the n-type poly-Si nanowire sensors. Figure 4(b) shows the schematic band diagram of the nanowire sensor to explain the I_on_ increase. The bottom-gate FETs' electrical behavior could be controlled by the substrate ionic concentration coupling induced the channel substrate potential modification [23]. The surface oxide coupling effect enables the nanowire substrate potential to have an opposite polarity from the ionic strength of the pH solution. The threshold voltage V_th_ of the poly-Si nanowire FETs determines the current flow in the nanowire channel, which can be described as in [24]:

(2) $$V_{th}=V_{0}-\Psi (pH)$$

where ψ(*pH*) is the effective substrate-coupling electrical potential-induced threshold modification, which is a function of pH. The final value of the potential can be expressed in a sensitivity factor, resulting in the following simple equation:

(3) $$\Psi =\gamma \times \left(\sqrt{pH_{\text{pzc}}}-\sqrt{pH}\right)$$

pH_pzc_ is the value of the pH for which the nanowire substrate surface is electrically neutral. The sensitivity factor γ determines the final sensitivity. The distinct pH sensitivity slope (V_th_ shift > 100 mV/pH) of the nanowire FETs follows the I_on_ increase (approximately 10%/pH).

**Figure 5.**
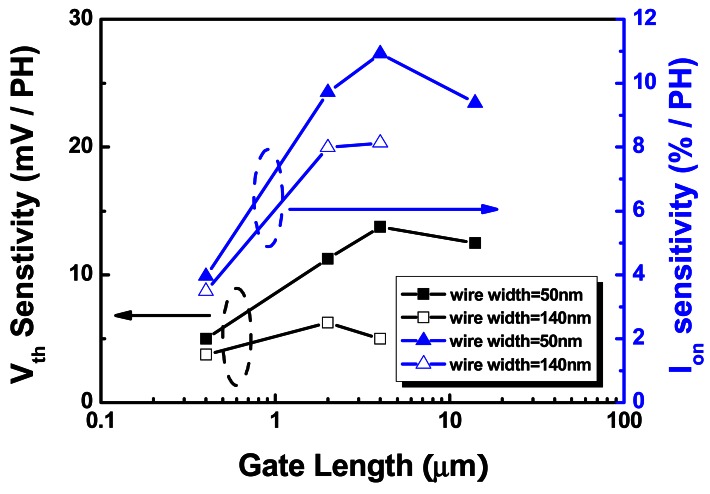
The pH sensitivity of poly-Si nanowire FETs sensor responses for various nanowire geometries.

This work also evaluates the pH sensitivity of the poly-Si nanowire FET sensor responses at various nanowire geometries. Figure 5 shows the driving current sensitivity and V_th_ shift with different pH solutions in various nanowire widths and channel length conditions. In the nanowire width direction, narrower width devices have higher pH sensitivity, which is consistent with most previous studies [25]. However, a short channel split did not show apparent pH sensitivity in the experiment of this paper. The short channel devices had a smaller intrinsic channel area, and the rest of the nanowire was doped with a heavy S/D dopant to reduce parasitic resistance of the nanowire and the influence of noise. The channel-coupling effect is attenuated at the heavy-doped nanowire region, thereby reducing the pH sensitivity of the short channel nanowire. Although short channel devices have large current shifts in different pH solutions (Figure 4(a)), the higher conduction current from the short channel device loses the nanowire potential control ability and pH sensitivity. Thus, the proper geometrical design to optimize device sensitivity and noise tolerance is a crucial consideration in the improvement of detection sensitivity in nanowire FET sensors.

**Figure 6.**
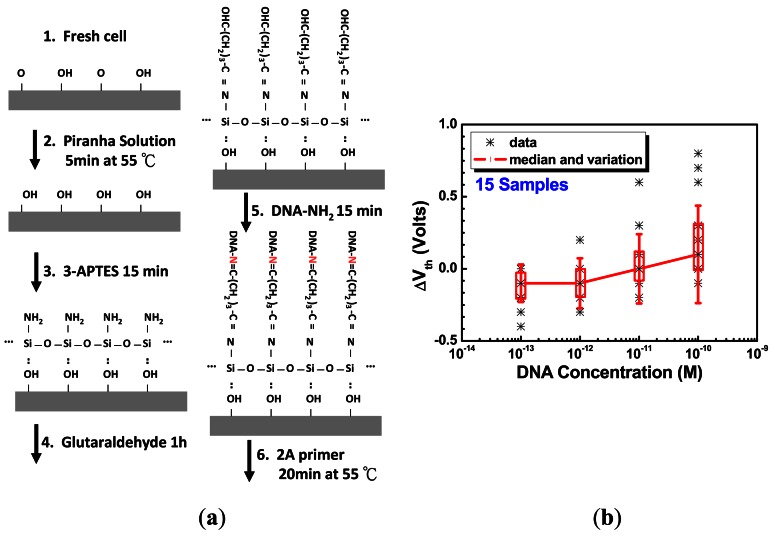
(**a**) Schematic representations of the nanowire surface after functionalized treatment steps. After the series process, oligo DNA can be bound effectively on the nanowire surface to react with the subsequent primer DNA; (**b**) The V_th_ shift of the poly-Si nanowire FETs at various DNA concentrations. Approximately 100 mV of the V_th_ shift is still present as the primer DNA concentration is lowered to 10 pM.

The primer DNA detection of the poly-Si nanowire FET sensors is also tested in this work. Figure 6(a) shows a schematic illustration of the poly-Si nanowire surface treatment steps for DNA concentration detection. First, the fresh nanowire surface was washed with piranha solution for 5 min to form a uniform OH bond. Subsequently, the 3-APTES solution was coated to link glutaraldehyde. After the series treatment process, the oligo DNA could be bound effectively on the nanowire surface to react with subsequent primer DNA. The V_th_ shift of the poly-Si nanowire FETs at various primer DNA concentrations is shown in Figure 6(b). Every condition indicates the median of the 15 devices at the same wafer, and the standard deviation is also shown. Approximately 100 mV of the V_th_ shift is still present as the primer DNA concentration is lowered to 10 pM. The concentration sensitivity is substantially higher than the conventional PCR limitation. This result demonstrates that the poly-Si nanowire FET sensors have high potential for DNA detection and application in gene engineering.

### 4.2. Nanowire as Stacked Memory

**Figure 7.**
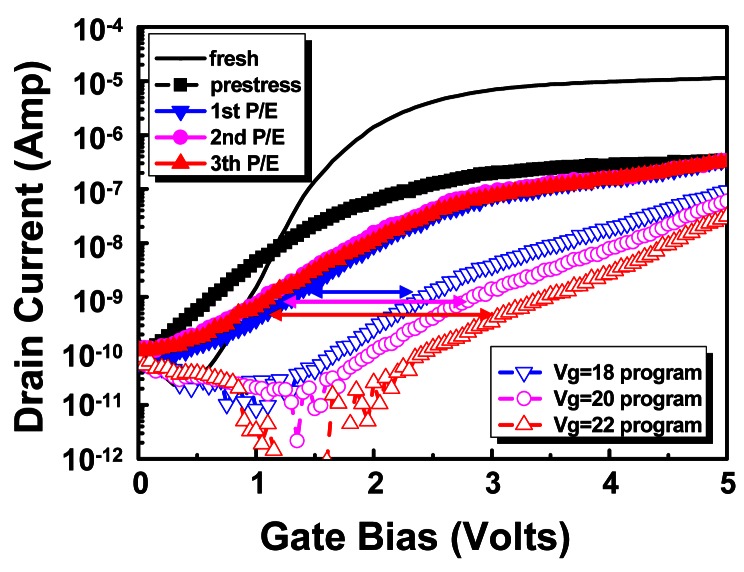
I_d_-V_g_ characteristics of the bottom-gate poly-Si nanowire FETs with ONO-buried oxide at different operation modes. With the completion of the initial pre-stress, V_th_ can be adjusted after adequate programming/erasing.

Specially designed ONO-buried oxide of the poly-Si nanowire was realized to obtain the charge storage layer of the memory application. In a high dielectric field, the nitride trap charge of the ONO-buried oxide can be filled or removed, and subsequently, the nanowire substrate channel potential would be modified. Figure 7 plots the I_d_-V_g_ characteristics of the ONO-buried oxide poly-Si nanowire FETs at different operation modes in a single nanowire device. After a short initial high negative gate bias pre-stress, the I_d_-V_g_ curve of the poly-Si nanowire FETs can be shifted to a relatively low V_th_ level because the trap charge of the buried oxide was neutralized. The I_d_-V_g_ curve can be erased effectively to the steady V_th_ level through a short erase pulse (10 ms), and V_th_ can be adjusted after achieving an adequate programming condition. Furthermore, the electrical characteristics of various operation conditions are reappearance.

**Figure 8.**
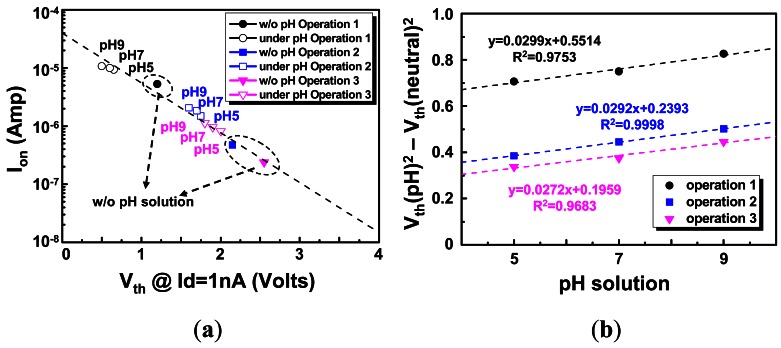
(**a**) I_on_-V_th_ distribution of the poly-Si nanowire FETs at various V_th_ levels. A universal curve is present in I_on_-V_th_ transformation with different operation conditions; (**b**) The normalized V_th_^2^ verse pH sensitivity of the poly-Si nanowire FETs at various operating conditions of V_th_.

The pH testing of the ONO-buried oxide poly-Si nanowire FETs was also implemented at various V_th_ levels. First, we recorded the initial V_th_ level and operated the pH testing at the initial state, designated operation 1 (at erase state, V_g_ = −12 V). Subsequently, the ONO-buried oxide nanowire device was programmed to a high V_th_ level, and operated the pH testing at a high V_th_ state, designated operation 2 (at program state, V_g_ = 18 V). Finally, a higher programming bias, designated operation 3 (at program state, V_g_ = 20 V), was operated in the same device to obtain a higher V_th_ level, and preceded pH testing. Figure 8(a) shows that the V_th_ shifts of surface pH ionic coupling and nitride charge trapping follow the same I_on_-V_th_ trend. Its pH sensitivity at a normalized scale V_th_(pH)^2^-V_th_(neutral)^2^ is plotted in Figure 8(b). Similar pH sensitivity slopes are in different V_th_ levels, and they are consisten with a previous pH ionic substrate-coupling model, as shown in Equation (3). The strongly V_th_-adjustable nanowire device provides a consistent electrical response for self-alterable correction and memory-integrated application.

**Figure 9.**
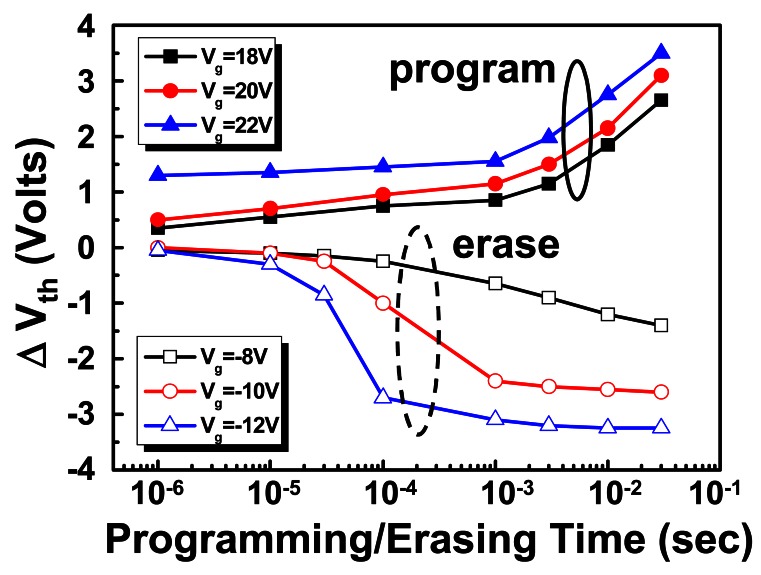
Programming/erasing efficiency characteristics of the ONO-buried oxide poly-Si nanowire FETs. The V_th_ shift can be larger than 3 V at the adequate operation bias because programming/erasing time is approximately 10 ms.

**Figure 10.**
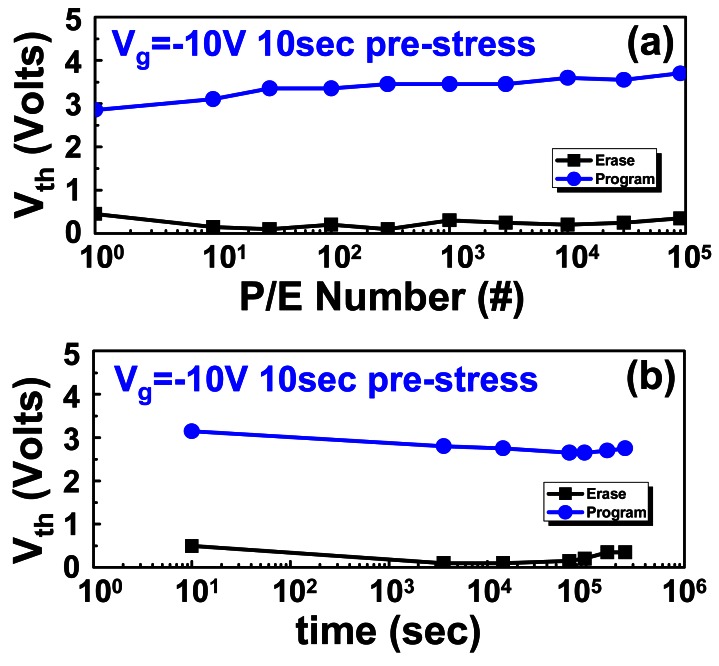
Endurance and retention characteristics of the ONO-buried oxide poly-Si nanowire FETs. The programming/erasing window is still larger than 2 V after 100 K programming/erasing operation cycles or a long storage period of 3 days.

Figure 9 shows a comparison of the programming and erasing efficiency characteristics of the ONO-buried oxide poly-Si nanowire FETs. The V_th_ shifts are over 3 V when programming/erasing time is approximately 10 ms at the adequate operation bias. Its endurance characteristics are shown in Figure 10(a). The programming and erasing V_g_ bias conditions are 20 V and −12 V, respectively. The programming/erasing cycles is shown to be more than 10^5^ operation cycles with an acceptable memory V_th_ programming/erasing window. The data retention characteristics are also shown in Figure 10(b). The memory programming/erasing window is still larger than 2 V after a long storage period of 3 days. These reliability characteristics both demonstrate that the ONO-buried oxide poly-Si nanowire FETs have great potential as the EEPROM cell in embedded memory applications. This approach to V_th_-adjustable nanowire FETs provides a possible System-on-Chip solution for self-alterable correcting and *in situ* recording.

Figure 9 shows a comparison of the programming and erasing efficiency characteristics of the ONO-buried oxide poly-Si nanowire FETs. The V_th_ shifts are over 3 V when the programming/erasing time is approximately 10 ms at the adequate operation bias. Its endurance characteristics are shown in Figure 10(a). The programming and erasing V_g_ bias conditions are 20 V and −12 V, respectively. The programming/erasing cycles is shown to be more than 10^5^ operation cycles with an acceptable memory V_th_ programming/erasing window. The data retention characteristics are also shown in Figure 10(b). The memory programming/erasing window is still larger than 2 V after a long storage period of 3 days. These reliability characteristics both demonstrate that the ONO-buried oxide poly-Si nanowire FETs have great potential as the EEPROM cell in embedded memory applications. This approach to V_th_-adjustable nanowire FETs provides a possible System-on-Chip solution for self-alterable correcting and *in situ* recording.

## 5. Conclusions

In conclusion, this paper reports a V_th_-adjustable nanowire biosensor integrated with a sensor/memory/CMOS application in a fully semiconducting process that provides a possible solution for the realization of System-on-Chip IC fabrication. The nanowire FETs have a distinct pH-sensitive slope (V_th_ shift > 100 mV/pH) and sensible DNA concentration detection (V_th_ shift > 100 mV in 10 pM). The strongly V_th_-adjustable nanowire FETs of the oxide-nitride-oxide buried oxide provide a consistent electrical response for self-alterable correction. Furthermore, the specially designed oxide-nitride-oxide buried oxide nanowire also demonstrates robust endurance and retention characteristics (>2 V programming/erasing window after 100 K programming/erasing cycles or 3-day storage) as embedded non-volatile memory application.

## Figures and Tables

**Table 1.** Comparisons of Si nanowire FETs made in previous studies and this work.

**Ref.**	**NW formation**	**NW material**	**NW uniformity**	**Process complexity**	**Fabrication cost**	**CMOS compatibility**	**pH sensitivity**
bottom-up [4]	CVD	Si	high	×	×	×	10%/pH
bottom-up [5]	RIE(Dry)	SOI	high	simple	×	×	-
bottom-up [6]	TMAH(Wet)	SOI	high	×	×	good	20×/pH
bottom-up [13]	E-beam	SOI	×	×	×	good	7×/pH
bottom-up [14]	E-beam	SOI	×	simple	×	good	15%/pH
bottom-up [10]	Spacer	poly-Si	×	simple	cheap	good	-
This work	BEOL	poly-Si		simple	cheap	good	10%/pH
